# The effect of mepivacaine on swine lingual, pulmonary and coronary arteries

**DOI:** 10.1186/s12871-015-0085-x

**Published:** 2015-07-14

**Authors:** Kenichi Satoh, Mami Chikuda, Ayako Ohashi, Miho Kumagai, Masahito Sato, Shigeharu Joh

**Affiliations:** 1 Division of Dental Anesthesiology, Department of Reconstructive Oral and Maxillofacial Surgery, School of Dentistry, Iwate Medical University, 1-3-27 Chuo-dori, Morioka, Iwate 020-8505 Japan; 2 Division of Special Care Dentistry, Department of Developmental Oral Health Science, School of Dentistry, Iwate Medical University, 1-3-27 Chuo-dori, Morioka, Iwate 020-8505 Japan

**Keywords:** Local anesthetic, Isometric tension, Vasocontractive activity, Swine artery

## Abstract

**Background:**

Although mepivacaine has a known biphasic action on the aortic and coronary artery in several animal species, its effects on the lingual and pulmonary artery are not well understood and it is not yet known whether mepivacaine produces vasoconstriction in these vessels. The present study aims to investigate the direct effects of mepivacaine on swine lingual, pulmonary and coronary arterial endothelium-denuded rings.

**Methods:**

Artery rings were perfused with isotonic 40 mM KCl until a stable constricted plateau was reached. The rings were then perfused with isotonic 40 mM KCl plus a particular concentration of mepivacaine (0.4 μM, 4.0 μM, 40 μM, 0.4 mM and 4.0 mM). The isometric tension strengths in each experiment were normalized to the strength of the isometric tension immediately before mepivacaine perfusion and expressed as a percentage.

**Results:**

Mepivacaine at 0.4 to 40 μM did not significantly alter 40 mM KCl-induced contraction in the lingual, pulmonary and coronary arterial rings. In contrast, mepivacaine at 4 mM produced attenuated vasoconstriction in the lingual, pulmonary and coronary arterial compared with isotonic 40 mM KCl.

**Conclusions:**

Mepivacaine produced vasoconstriction at lower concentrations, followed by attenuated vasoconstriction at higher concentrations on swine lingual, pulmonary and coronary arterial endothelium-denuded rings. Mepivacaine (4 μM) appeared to increase isotonic 40 mM KCl-induced contraction, followed by attenuated vasoconstriction at 4 mM. Dentists using 3 % mepivacaine should take into consideration that the risk of complications may be increased if more than six mepivacaine cartridges are used in dental treatment or minor surgery, or if over 15 ml of mepivacaine is administered to a patient with cardiovascular complications during general anesthesia for oral maxillofacial surgery.

## Background

Amino-amide local anesthetics including mepivacaine, lidocaine, ropivacaine and levobupivacaine have a biphasic action on the smooth muscle of peripheral vessels, ranging from vasoconstriction to vasodilation depending on the concentration used [3, 11, 16]. Mepivacaine essentially has mild vasoconstrictive activity in vessels [15]. Although mepivacaine has a biphasic action on the aortic or coronary artery in several animal species [12, 18, 20], there are few studies examining the effects of mepivacaine on other peripheral vessels, such as the craniofacial arteries or the pulmonary artery.

Dentists typically use mepivacaine without vasoconstrictors for short-duration dental procedures and oral maxillofacial surgery. Regarding absorption rates and blood kinetics of mepivacaine, the maximum concentration in plasma was 4.65 ± 0.47 μg/ml 15 min after administration of 25 ml of 2 % mepivacaine to epidural blocks, and 3.53 ± 0.39 μg/ml 25 min after administration of 25 ml of 2 % mepivacaine to peripheral nerve brachial plexus blocks [19]. In dental treatment, mean peak serum levels of 0.69 μg/ml were reached when one dental cartridge (1.8 ml) containing 54 mg mepivacaine was infiltrated at the mucosa on the apex of the right maxillary second bicuspid, while the mean peak levels were 1.31 μg/ml when two dental cartridges (3.6 ml) containing a total of 108 mg mepivacaine were infiltrated at the same mucosa [6]. The rate and degree of diffusion is governed by the degree of plasma protein binding, the degree of ionization, and the degree of lipid solubility. Local anesthetics appear to be inversely related to the degree of plasma protein binding, as only the free, unbound drug is available; mepivacaine is approximately 77 % bound to plasma proteins [2]. The most commonly encountered acute adverse experiences which demand immediate countermeasures are related to the central nervous system and the cardiovascular systems. These adverse experiences are generally dose-related and due to high plasma levels. In additional to systemic dose-related toxicity, an intentional subarachnoid injection of drug during the intended performance of caudal or lumbar epidural block or nerve blocks near the vertebral column may result in underventilation or apnea. Toxic blood concentrations depress cardiac conduction and excitability, which may lead to atrioventricular block and ultimately to cardiac arrest [17].

The effects of mepivacaine on local regions such as craniofacial and pulmonary arteries are not well-known. Mepivacaine has such a diversity of effects on smooth muscle that we cannot guess its effects on any particular smooth muscle. Although mepivacaine has a biphasic action on the aortic or coronary artery in several animal species, its effects on the lingual or the pulmonary artery are not well understood. It has also not yet been fully investigated whether mepivacaine produces vasoconstriction in these vessels, and if mepivacaine *does* produce vasoconstricion in these vessels, the concentrations producing vasoconstriction have not yet been determined. The present study aims to clarify these issues. Thus, the present study was designed to further investigate the direct effects of mepivacaine on these vessels *in vitro* using swine lingual and pulmonary arterial rings and compared with the coronary artery.

## Methods

This study was approved by the Institutional Review Committee on the Ethics of Animal Experiments of Iwate Medical University. All experiments were conducted in accordance with the Institutional Animals Care and Use Committee guidelines.

### Reagents and solutions

Mepivacaine hydrochloride was purchased from Sigma–Aldrich Corp. (St. Louis, USA). All other chemicals were obtained from Wako Pure Chemical Industries (Osaka, Japan). In all experiments, air-equilibrated Hank’s balanced salt solution (HBSS) was used to maintain the arteries under resting conditions. HBSS was made up of 137 mM NaCl, 5.4 mM KCl, 0.8 mM MgSO_4_, 1.26 mM CaCl_2_, 0.34 mM Na_2_HPO_4_, 0.44 mM KH_2_PO_4_, 4.2 mM NaHCO_3_, and 5.55 mM glucose (pH 7.34). All other salt solutions used as perfusate were made by modifying HBSS. Isotonic 40 or 100 mM KCl solution was prepared by replacing the NaCl in the HBSS solution with an equimolar amount of KCl. Each isotonic 40 mM KCl alone and isotonic 40 mM KCl plus mepivacaine treatment was made by adding the component agents into the isotonic 40 mM KCl immediately before use.

### Artery ring preparation and isometric tension measurement

Fresh swine tongues, lungs and hearts were obtained from a local abattoir. A segment of the lingual artery at the proximal region of the tongue, third generation pulmonary artery and left coronary artery was dissected out. After the adventitia was removed, the lingual artery and coronary artery segments (approximately 2 mm in diameter) and the pulmonary artery segments (approximately 2–3 mm in diameter) were cut into 3-mm-long rings, and the lumen surface was rubbed gently against a thin arm of stainless steel tweezers to remove the endothelium [7]. It was confirmed that 3 μM acetylcholine-induced relaxation of the artery rings preconttracted with 40 mM KCl. The artery rings were kept in HBSS at 5 °C until used for measurement.

An artery ring was held with two tungsten needles in the perfusion chamber (containing 3 ml of perfusate). One needle was fastened to a displacement transducer (Type UL-2GR, Minebea Co., Fujisawa, Japan), and the other to a micromanipulator. The solution was bubbled with a mixture of 95 % O_2_ and 5 % CO_2_, and held at a temperature of 37 °C and flow rate of 1.6 ml/min with a peristaltic pump (SMP-23, Tokyo Rikakikai Co., Tokyo, Japan). As the strength of contraction did not change when the resting tone was 3–7 mN, the artery rings were extended to give a resting tone of approximately 5 mN and immediately tested for contractility by two 2.5 min perfusions with isotonic 100 mM KCl separated by a 10 min HBSS perfusion. After a 30 min HBSS perfusion, artery rings were perfused with isotonic 40 mM KCl. When a stable constricted plateau was reached with isotonic 40 mM KCl, an isotonic 40 mM KCl solution containing mepivacaine at concentrations of 0.4 μM, 4.0 μM, 40 μM, 0.4 mM and 4.0 mM was perfused. Each concentration was perfused for 5 min. HBSS perfusion was always performed after all drug perfusions were completed. Isometric tension was detected using a displacement transducer, and signals detected were amplified with a carrier amplifier (CSD-815 Digital indicator, Minebea Co., Fujisawa, Japan) and recorded with a Powerlab 16/30 T data acquisition system (ADInstruments, Bella Vista, Australia). The isometric tension strengths in each experiment were normalized to the strength of the isometric tension immediately before mepivacaine perfusion. Vascular response induced by mepivacaine in arterial rings was expressed as the percentage of the contraction induced by isotonic 40 mM KCl.

### Statistical analyses

Dilation was determined by measuring the cumulative reduction in induced tone in the arterial segments, and was expressed as the percentage of the contraction induced by isotonic 40 mM KCl. A value of 0 % indicates initial resting tension (5 mN), and a value of 100 % indicates the isometric tension generated by exposure to isotonic 40 mM KCl. Values greater than 100 % indicate that contraction occurred in response to mepivacaine. Values are presented as mean ± standard deviation. Statistical analysis was performed using SPSS, version 11.0 (SPSS, Chicago, IL, USA). The Shapiro-Wilk test was used for normality, and Bartlett’s test was used for homogeneity of variance. Repeat measure analysis of variance (ANOVA) followed by Bonferroni’s post-test was performed. Differences were considered significant at P < .05.

## Results

**Fig. 1:**
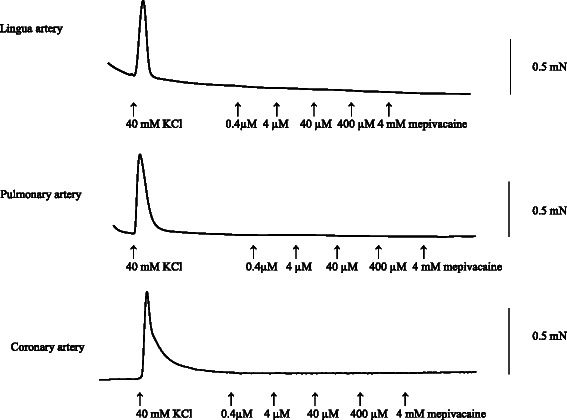
Effect of mepivacaine on arterial rings under resting conditions in Hank’s balanced salt solution. There was no response induced by mepivacaine in lingual, pulmonary and coronary arterial rings

**Fig. 2:**
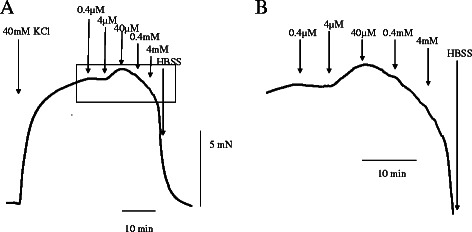
A typical trace showing the effect of mepivacaine on lingual artery ring contraction induced with 40 mM KCl. **a** Representative recording of the mepivacaine-induced contraction. **b** Enlarged view of a square portion of (**a**)

**Fig. 3:**
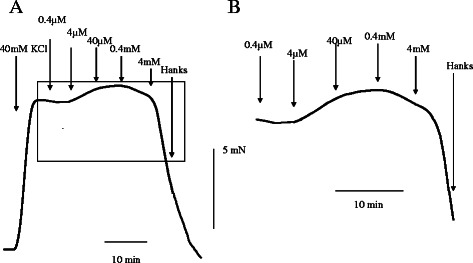
A typical trace showing the effect of mepivacaine on pulmonary artery ring contraction induced with 40 mM KCl. **a** Representative recording of the mepivacaine-induced contraction. **b** Enlarged view of a square portion of (**a**)

**Fig. 4:**
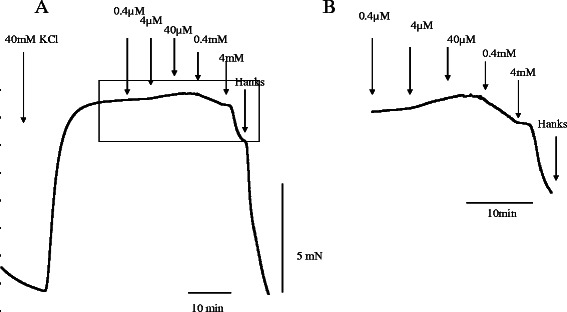
A typical trace showing the effect of mepivacaine on coronary artery ring contraction induced with 40 mM KCl. **a** Representative recording of the mepivacaine-induced contraction. **b** Enlarged view of a square portion of (**a**)

**Fig. 5:**
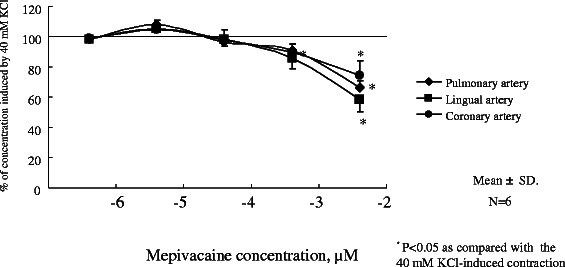
Dose-dependent response of mepivacaine on lingual, pulmonary and coronary vasorelaxation. Endothelium-denuded lingual, pulmonary and artery rings were challenged with a cumulative dose of mepivacaine. Tension was determined by isometric force transduction, and is expressed as a percentage of the 40 mM KCl-induced contraction (*N* = 6). N indicates the number of arterial rings

There was no response induced by mepivacaine in lingual, pulmonary and coronary arterial rings under resting conditions in HBSS (Fig. 1). Mepivacaine resulted in dilation of the swine lingual, pulmonary and coronary arterial rings that were maintained in a stable, constricted state; typical traces of the changes in isometric tension in response to increasing mepivacaine concentrations (0.4 μM to 4 mM) are shown in Figs 2, 3 and 4. Dose response curves at these concentrations are shown in Fig. 5. Mepivacaine at 0.4 to 40 μM did not significantly alter 40 mM KCl-induced contraction in the lingual, pulmonary and coronary arterial rings (Table 1). In contrast, the highest concentration of mepivacaine (4 mM) produced attenuated vasoconstriction. There were significant differences between the effects of the control solution (40 mM KCl) and the effect of 4 mM mepivacaine on the lingual artery, 400 μM and 4 mM mepivacaine on the pulmonary artery, and 4 mM mepivacaine on the coronary artery.

**Table 1:** Effects of mepivacaine on lingual, pulmonary and coronary artery

Mepivacaine (μM)	Lingual artery (%)	Pulmonary artery (%)	Coronary artery (%)
40 mM KCl	100	100	100
0.4	98.3 ± 1.4	98.3 ± 1.2	99.0 ± 0.9
4	108.2 ± 2.6	105.1 ± 2.6	103.1 ± 1.1
40	96.6 ± 6.4	98.1 ± 3.0	98.0 ± 4.6
400	90.6 ± 4.6	85.8 ± 5.2^*^	90.0 ± 6.8
4000	66.1 ± 10.2^*^	58.5 ± 5.0^*^	74.1 ± 8.2^*^

Data are mean ± SD. Values are expressed as the percentage of contraction induced by isotonic 40 mM KCl

*P<0.05 as compared with the 40 mM KCl-induced contraction

## Discussion

Mepivacaine produced vasoconstriction at lower concentrations, followed by attenuated vasoconstriction at higher concentrations on swine lingual, pulmonary and coronary arterial endothelium-denuded rings under isotonic conditions. Numerous studies have demonstrated that amino-amide local anesthetic agents can exhibit biphasic vascular effects, with contraction at low concentrations and decreased contraction or even dilation at high concentrations reported in aortic or coronary arterial rings in several animal species [4–6]. The biphasic vascular effect is a common characteristic of amino-amide local anesthetic agents that has been extensively studied [1, 4, 10]. However, biphasic activity has been largely reported in rat aortic or coronary arteries, and to our knowledge there are no studies examining the effects of mepivacaine on craniofacial arteries such as the lingual artery. In this study, we found that mepivacaine appeared to increase isotonic 40 mM KCl-induced contraction at 4 μM concentration, caused dilation at higher concentrations from 40 μM to 4 mM in the lingual and coronary artery, and dose-dependent dilation from 40 μM to 4 mM in the pulmonary artery. We did not investigate the mechanism(s) of smooth muscle constriction and dilation by mepivacaine in this study. A previous report investigating the effect of mepivacaine on the isolated rat aorta found that verapamil and calcium-free Krebs solution attenuated mepivacaine-induced contraction of the endothelium-denuded aorta [16]. Thus, calcium influx via voltage-operated calcium channel (VOCC) activation by low concentrations of mepivacaine may trigger the initial contraction, while a high concentration of mepivacaine had an inhibitory effect on the VOCCs in vascular smooth muscle [16].

Mepivacaine (4 μM) appeared to increase isotonic 40 mM KCl-induced contraction, followed by attenuated vasoconstriction at 4 mM compared with isotonic 40 mM KCl-induced contraction. Similarly, a previous study found that mepivacaine produced contraction at low concentrations (1 × 10^−3^ and 3 × 10^−3^ mol/L) followed by dilation at a high concentration (1 × 10^−2^ mol / L) in the rat aorta [16]. It has been reported that contraction induced by KCl and phenylephrine is attenuated in the presence of mepivacaine (10^−3^ M) in isolated endothelium-denuded rat aorta [10]. In contrast, lower concentrations of mepivacaine (10^−9^–10^−5^ M) did not evoke any changes in tension in isolated ciliary artery rings [8]. Furthermore, it was reported that in response to transmural stimulation, to the tension of rabbit aortic strips was not altered by mepivacaine in concentrations raging from 5 × 10^−4^ to 2 × 10^−3^ M [9]. In the present study, administration of 4.0 μM mepivacaine appeared to increase isotonic 40 mM KCl-induced contraction in swine lingual, pulmonary and coronary arterial rings, which is a lower concentration than those previously reported. The reasons for these differences in the mepivacaine concentration are unknown, but may relate to differences in species and location [16].

Our previous study suggested that lidocaine at low concentrations produced vasoconstriction in swine lingual and pulmonary arteries, while vasodilation was seen at higher lidocaine concentrations of 1 μg/ml [14]. Perlmutter reported that a lidocaine concentration of 10 μg/ml caused mild vasoconstriction in the porcine coronary artery [12]. Therefore we used mepivacaine at 0.4 μM, 4.0 μM, 40 μM, 0.4 mM and 4.0 mM concentrations as these are almost equivalent to 0.11 μg/ml, 1.1 μg/ml, 11 μg/ml, 110 μg/ml, and 1,100 μg/ml concentrations.

The circulating levels of mepivacaine after dental injections have been previously studied. Mean peak serum levels of 0.69 μg/ml were reported when one dental cartridge containing 54 mg mepivacaine was infiltrated at the mucosa on the apex of the right maxillary second bicuspid, while the mean peak levels were 1.31 μg/ml when two dental cartridges containing 108 mg mepivacaine were infiltrated at the same mucosa [6]. It is thought that the concentration of mepivacaine encountered in the clinical setting is approximately 77 % protein-bound, and the rest is free in plasma. If approximately 77 % of lidocaine is bound to plasma proteins, we would assume that only the 23 % free in plasma would affect vascular muscle directly [2]. Hence, when one dental cartridge containing 54 mg mepivacaine is infiltrated into the mucosa, there would presumably be mean peak levels of 0.16 μg/ml free in plasma. In the present study, 4 μM (1.1 μg/ml) mepivacaine appeared to increase isotonic 40 mM KCl-induced contraction in swine lingual, pulmonary and coronary arterial rings. Thus, if we consider the amount of free plasma mepivacaine when dentists infiltrate six to eight dental mepivacaine cartridges into the oral mucosa, mepivacaine-induced vasoconstriction may occur in the orofacial, pulmonary and coronary arteries. An increase in the plasma concentration of mepivacaine would increase the concentration of free plasma mepivacaine. Therefore, we think it is possible that mepivacaine-induced vasoconstriction may occur in orofacial and pulmonary arteries when less than six to eight mepivacaine cartridges are infiltrated into the oral mucosa. In dental treatment, we usually use one or two 3 % mepivacaine cartridges, but occasionally more than six cartridges are used for minor surgery or extraction. Dentists are educated to avoid using lidocaine with adrenaline cartridges for patients with cardiovascular complications, especially with a past history of vasospasm, hypertension, and heart failure; therefore in these patients local anesthetic without adrenaline is used, for example mepivacaine or prilocaine with felypressin, to prevent side effects induced by adrenaline. In patients with cardiovascular complications, if more than six cartridges of 3 % mepivacaine are used in dental treatment or minor surgery, or over 15 ml at a time during general anesthesia for oral maxillofacial surgery, this could potentially increase the risks of complication by mepivacaine-induced vasoconstriction in the lingual, coronary and pulmonary arteries. In addition, mepivacaine-induced contraction may be attenuated in patients taking calcium channel antagonists for the management of hypertension, leading to a short duration of mepivacaine-induced analgesia [13]. Mepivacaine-induced vasoconstriction may contribute to pale skin color, decreased cutaneous blood flow, attenuated capillary blood flow increase, attenuated reactive hyperthermia observed after analgesia induced by mepivacaine, and intermediate duration of mepivacaine-induced analgesia [16].

There are some limitations to our study. Amino-amide local anesthetics release nitric oxide, which produces vasodilation of isolated vessels [3, 11, 16]; hence, mepivacaine-induced vasoconstriction is attenuated in endothelium-intact vessels compared with endothelium-denuded vessels. As we used endothelium-denuded arterial rings in the current study, mepivacaine-induced vasoconstriction would be attenuated in an *in vivo* vessel with its endothelium intact. In addition, the data in this study are based on *n* = 6, which by all accounts is a small sample size. However, despite the small sample size and insufficient power analysis, we believe that the data provide accurate and reliable information on the effect of mepivacaine on arterial rings, and the findings are useful for further investigation of craniofacial arteries. Further research needs to be done to investigate whether our *in vitro* results correlate with real-life situations.

## Conclusions

This study showed that a low concentration of mepivacaine produced vasoconstriction, followed by attenuated vasoconstriction at higher concentrations on swine lingual, pulmonary and coronary arterial endothelium-denuded rings. It also showed that mepivacaine (4 μM) appeared to increase isotonic 40 mM KCl-induced contraction, followed by attenuated vasoconstriction at 4 mM compared with isotonic 40 mM KCl-induced contraction. Thus, dentists should consider that the risks of general complications are potentially increased if more than six cartridges of 3 % mepivacaine are used in dental treatment or minor surgery, or over 15 ml of mepivacaine is used at a time for a patient with cardiovascular disease during general anesthesia for oral maxillofacial surgery.

## Abbreviations

HBSS: Hank’s balanced salt solution
VOCC: Voltage-operated calcium channel
